# Limited Evidence for Selection at the *FADS* Locus in Native American Populations

**DOI:** 10.1093/molbev/msaa064

**Published:** 2020-03-07

**Authors:** Iain Mathieson

**Affiliations:** Department of Genetics, Perelman School of Medicine, University of Pennsylvania, Philadelphia, PA

**Keywords:** diet, selection, ancient DNA, FADS, PBS

## Abstract

The *FADS* locus contains the genes *FADS1* and *FADS2* that encode enzymes involved in the synthesis of long-chain polyunsaturated fatty acids. This locus appears to have been a repeated target of selection in human evolution, likely because dietary input of long-chain polyunsaturated fatty acids varied over time depending on environment and subsistence strategy. Several recent studies have identified selection at the *FADS* locus in Native American populations, interpreted as evidence for adaptation during or subsequent to the passage through Beringia. Here, we show that these signals are confounded by independent selection—postdating the split from Native Americans—in the European and, possibly, the East Asian populations used in the population branch statistic test. This is supported by direct evidence from ancient DNA that one of the putatively selected haplotypes was already common in Northern Eurasia at the time of the separation of Native American ancestors. An explanation for the present-day distribution of the haplotype that is more consistent with the data is that Native Americans retain the ancestral state of Paleolithic Eurasians. Another haplotype at the locus may reflect a secondary selection signal, although its functional impact is unknown.

Long-chain polyunsaturated fatty acids are essential for many aspects of cellular and organismal function (Marszalek and Lodish 2005; Darios and Davletov 2006). Although they can be obtained from dietary sources, they can also be synthesized from short-chain polyunsaturated fatty acid (SC-PUFA) through the ω-3 and ω-6 biosynthesis pathways. Some of the steps in these pathways are catalyzed by the fatty acid desaturase genes *FADS1* and *FADS2* (Nakamura and Nara 2004), which are located close to each other on human chromosome 11. This locus (which we refer to as the *FADS* locus) has been targeted by selection multiple times in human evolution (Ameur et al. 2012; Mathias et al. 2012; Mathieson et al. 2015; Buckley et al. 2017; Ye et al. 2017; Mathieson and Mathieson 2018). There are two LD blocks at the locus, but most studies have focused on the two major haplotypes at LD block 1 (Ameur et al. 2012), which we refer to as the *ancestral* (A) and *derived* (D) haplotypes. Haplotype D increases expression of *FADS1* and is hypothesized to be an adaptation to a diet relatively low in PUFA, whereas haplotype A is hypothesized to be advantageous in a PUFA-rich environment (Ameur et al. 2012; Fumagalli et al. 2015). LD block 1 is also where the strongest genome-wide association study signals for lipid levels are detected in European ancestry populations (Teslovich et al. 2010). Haplotype D appears to have been under selection in Africa—indeed, this selection may have preceded the out-of-Africa bottleneck (Mathias et al. 2012; Mathieson and Mathieson 2018)—and it is virtually fixed in present-day African populations (Ameur et al. 2012; Mathias et al. 2012). Given this, it is surprising that early Eurasian populations appear to have largely carried the ancestral haplotype, suggesting selection for the ancestral haplotype at some time after the split of present-day African and non-African lineages (Ye et al. 2017; Mathieson and Mathieson 2018). By the Mesolithic—around 10,000 years before present (BP)—the ancestral haplotype was fixed in Europe (Mathieson et al. 2015). The derived haplotype was reintroduced to Europe in the Neolithic (around 8,400 BP) by the migration of Early Farming populations from Anatolia (Mathieson et al. 2015), experienced strong positive selection during the Bronze Age (Buckley et al. 2017; Mathieson and Mathieson 2018), and is now at a frequency of around 60%. The trajectory of the derived haplotype in East Asian populations is less clear. It is common today in East Asia (∼40%), and the locus does exhibit a signal of selection in East Asian populations (Suo et al. 2012; 1000 Genomes Project Consortium 2015; Chiang et al. 2018). But, with limited ancient DNA evidence, it is unclear whether this represents recent selection or ancient shared ancestry with African lineages.

## The Ancestral Haplotype Was Common in Upper Paleolithic Eurasia

**Fig. 1:**
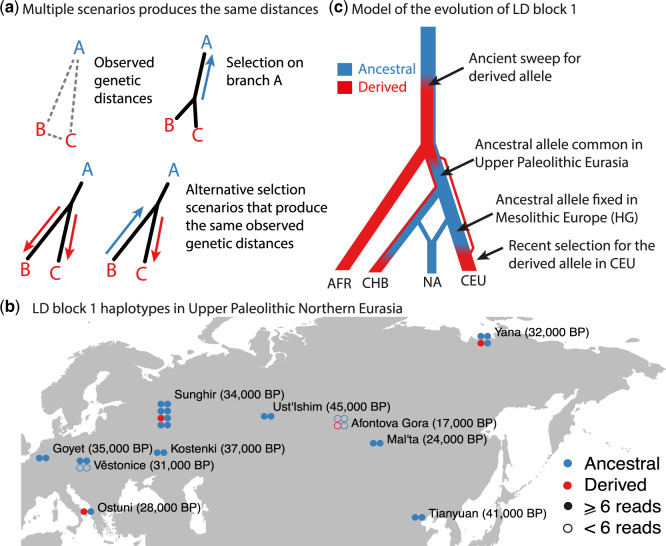
(*a*) The PBS compares genetic differentiation (branch length) between three populations. If a new mutation (blue) is under selection in one population (A), the branch leading to A will be longer—a signal of selection. If a haplotype that exists in the ancestral population (red) is under selection in both populations B and C then, because B and C look very similar, the PBS misattributes the long branch to A, instead of to B and C. (*b*) LD block 1 haplotypes in Upper Paleolithic Eurasia (Fu et al. 2014, 2016; Raghavan et al. 2014; Sikora et al. 2017, 2019; Yang et al. 2017). Ancestral and derived haplotype defined as haplotypes A and C (Mathieson and Mathieson 2018). (*c*) Model for the evolution of present-day African (AFR), East Asian (CHB), Native American (NA), and European (CEU) population showing where derived (red) and ancestral (blue) haplotypes are common.

We examined ancient DNA from 16 individuals from Early Upper Paleolithic Eurasia (Fu et al. 2014, 2016; Raghavan et al. 2014; Sikora et al. 2017, 2019; Yang et al. 2017). Of these individuals’ 32 haplotypes, 4 are derived and 28 are ancestral (3 vs. 23 supported by more than 6 reads; fig. 1*b* and supplementary table 1, Supplementary Material online). This confirms that the derived LD block 1 haplotype was uncommon, though not completely absent, throughout Upper Paleolithic Eurasia. With a sufficiently intense bottleneck, genetic drift could fix the ancestral haplotype in the ancestors of Native Americans even if it was not under selection (Harris et al. 2019).

## Selection Scans at the Locus Are Confounded by Independent Adaptation

Excluding the effects of recent admixture, present-day Native American, Inuit, and Siberian populations are fixed for the ancestral haplotype (Ameur et al. 2012; Fumagalli et al. 2015; Harris et al. 2019). Fumagalli et al. (2015) found a strong signal of selection at the locus in the Greenlandic Inuit population, which they interpret as an adaption to a PUFA-rich Arctic diet at least 20,000 years ago in the common ancestors of present-day Inuit and Siberian populations. Subsequent studies detected a similar signal in Native American populations (Amorim et al. 2017; Harris et al. 2019). Some studies interpret this as evidence for selection for the ancestral haplotype relatively early on the Native American lineage (Hlusko et al. 2018), although it could also represent a shared signal from the population ancestral to both Native Americans and Inuit. Both these signals extend across LD blocks 1 (tagging the ancestral haplotype) and 2.

These studies used the population branch statistic (PBS) (Yi et al. 2010) to compare Native American (NA), European (EUR), and East Asian (EAS) populations. The PBS compares genetic differentiation between three populations and identifies which, if any, of the three branches has excess differentiation that indicates selection. We write PBS(A, (B, C)) to denote the statistic that is testing for excess differentiation on the A branch, relative to B and C. However, the PBS makes the implicit assumption that each of the three branches is independent. If there is parallel selection on the same haplotype in two population, say B and C, then B and C will be similar to each other, but each will be highly diverged relative to A. Thus, the PBS will misattribute selection to branch A (fig. 1*a*). Given low frequency of the derived allele in Upper Paleolithic Eurasia (fig. 1*b*) and high frequencies in present-day European and East Asian populations, we expect that PBS(NA, (EUR, EAS)) would give a spurious signal of selection in the Native American population (fig. 1*c*).

**Fig. 2:**
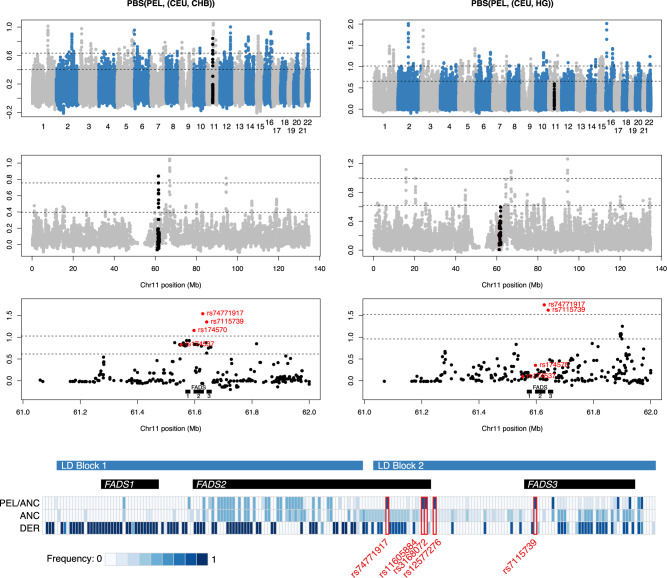
PBS on the Native American branch. Left column: PBS(PEL,(CEU, CHB)). Right column: PBS(PEL,(CEU, HG)). Upper row: genome-wide PBS in overlapping 20-SNP windows, shifted by 5 SNPs. Black points indicate the region Chr11:61-62Mb (hg19). Second row: chromosome 11 PBS in overlapping 20-SNP windows. Third row: per-SNP PBS in the region Chr11:61-62Mb. Horizontal lines indicate upper 0.01 and 0.001 genome-wide PBS quantiles. Red labeled points indicate SNPs previously identified as targets of selection (Fumagalli et al. 2015; Amorim et al. 2017). Top three rows restricted to 903,961 autosomal SNPs present on the 1,240k capture array with a minor allele frequency of at least 5% in at least one of the four populations. Fourth row: frequencies for all SNPs at >1% frequency in at least one population in CEU and CHB individuals carrying the derived haplotype (DER), the ancestral haplotype (ANC), and for PEL individuals carrying the ancestral haplotype (PEL/ANC). Color indicates the frequency of the variant that is rarer on the ancestral haplotype. Highlighted in red are five LD block 2 SNPs that have >50% difference in frequency between ANC and PEL/ANC, and at most 10% frequency in DER.

We tested this by computing the PBS using Native American, European, and Mesolithic European populations. We used the Peruvians from Lima (PEL) 1000 Genomes population to represent Native Americans (1000 Genomes Project Consortium 2015). For each PEL individual, we restricted to regions of homozygous Native American ancestry (Martin et al. 2017) (93 chromosomes, on average). We used CEU (Northern and Western European ancestry) to represent present-day Europeans, CHB (Chinese from Beijing) to represent East Asians and 150 ancient European hunter-gatherers (HG, 68 chromosomes, on average) to represent Mesolithic Europe (Skoglund et al. 2012; Gamba et al. 2014; Lazaridis et al. 2014; Olalde et al. 2014, 2018; Allentoft et al. 2015; Jones et al. 2015; Mathieson et al. 2015, 2018; González-Fortes et al. 2017; Lipson et al. 2017; Saag et al. 2017). We replicate the elevated PBS(PEL, (CEU, CHB)) statistic at the *FADS* locus (fig. 2 left column; upper 0.0002 quantile), but find that it largely disappears if we replace CHB with HG (fig. 2 right column; upper 0.016 quantile). We see similar results if we replace CEU instead of CHB (supplementary fig. 1, Supplementary Material online), or replace PEL with a diverse panel of 28 unadmixed Native American genomes from the Simons Genome Diversity Project (Mallick et al. 2016) (supplementary fig. 2, Supplementary Material online). Two of the LD block 2 single-nucleotide polymorphisms (SNPs) originally identified by Fumagalli et al. (2015)—rs74771917 and rs7115739—still have extreme PBS values, but PBS is no longer elevated across the locus (fig. 2). Conversely, both PBS(CEU, (PEL, HG)) and PBS(CHB, (PEL, HG)) do show elevated values (upper 0.00004 and 0.0006 quantiles; supplementary fig. 3, Supplementary Material online). These results are consistent with selection in CEU and either selection or retention of the derived haplotype in CHB.

## A Potential Secondary Signal of Selection

Although the Native American PBS signal in LD block 1 (containing rs174570, rs174556, and rs174537) disappears when Mesolithic HG are used as an outgroup, two SNPs (rs74771917 and rs7115739) in LD block 2 remain significant (fig. 2). We also identified three additional SNPs that are highly differentiated between present-day Native American and Eurasian ancestral allele carriers, using sequence data from the 1000 Genomes Project (fig. 2, lower panel). Among individuals who carry the ancestral haplotype at LD block 1, this LD block 2 haplotype has a frequency of around 100% in the Greenland Inuit (Fumagalli et al. 2015), 82% in PEL, 34% in CHB, 9% in HG, and 4% in CEU. The Anzick individual (Rasmussen et al. 2014) carries two copies, whereas the 40,000-year old East Asian Tianyuan individual (Yang et al. 2017) carries one. It therefore remains possible that the high frequency of this haplotype represents a secondary signal of selection in the common ancestor of Inuit and Native Americans. On the other hand, this region is not a genome-wide outlier in the standard window-based PBS analysis, and the frequency of this haplotype may have been driven by linked selection on LD block 1. Further, the LD block 2 haplotype has not been shown to affect expression of any of the *FADS* genes or any other phenotype, independent of the LD block 1 haplotype. Within-population, the two blocks are highly correlated, so it would be necessary to perform conditional analysis at the locus in East Asian populations to identify an independent effect of the block 2 haplotype.

## Conclusion

We demonstrated that the apparent signal of selection for the ancestral allele at the *FADS* locus in Native American populations could actually reflect high frequencies of the derived allele in Europe and East Asia. Direct evidence from ancient DNA demonstrates recent (Holocene) selection for the derived allele in Europe, whereas its high frequency in East Asia could be the result of either recent selection or more ancient shared ancestry. The ancestral haplotype at LD block 1 may have been selected in Upper Paleolithic Eurasian populations but this likely took place around or before the split of East and West Eurasian populations 40–60,000 years ago and certainly before the Native American and Siberian lineages split. There remains some evidence of a secondary signal of selection in LD block 2 but this is shared by Inuit and Siberians and not specific to Native Americans. The complex history of selection at this locus likely confounds selection scans in other populations as well. Finally, this analysis demonstrates a limitation of the commonly used PBS selection test, and the ability of direct evidence from ancient DNA to resolve complex evolutionary histories that may not be identifiable using present-day data.

## Supplementary Material

Supplementary data are available at *Molecular Biology and Evolution* online.

## Supplementary Material

msaa064_Supplementary_Data
