# Supplementary material for: Limited Evidence for Selection at the FADS Locus in Native American Populations
Source: Mol Biol Evol. 2020 Mar 7;37(7):2029–33. doi: 10.1093/molbev/msaa064 (PMC7306688; doi:10.1093/molbev/msaa064)
Supplement: msaa064_Supplementary_Data [file msaa064_supplementary_data.pdf]

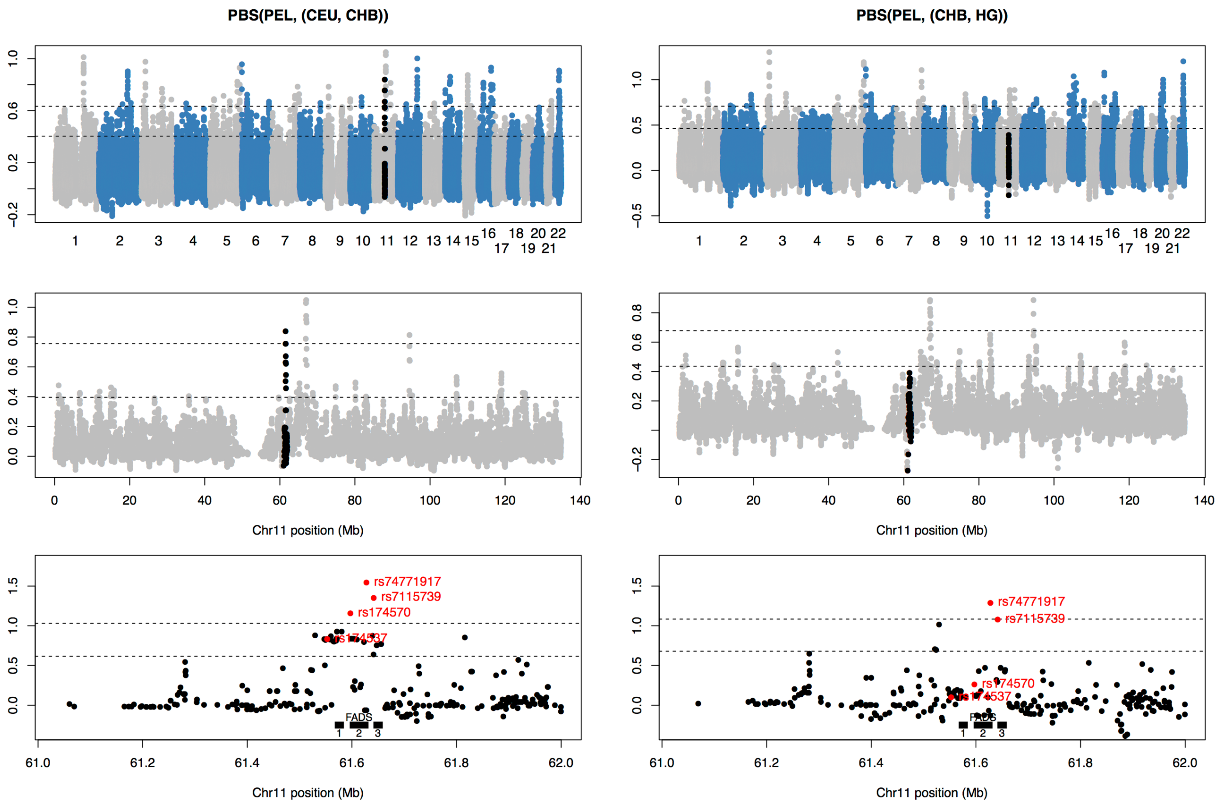

Supplementary Figure 1: As Figure 2, but with PBS(PEL,(CHB,HG)) in the right-hand column, instead of PBS(PEL, (CEU,HG).

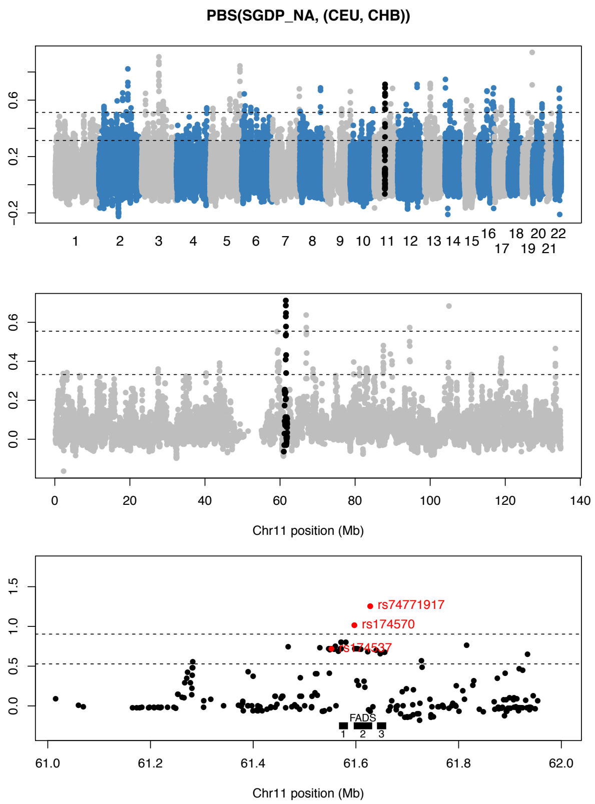

Supplementary Figure 2: As Figure 2, but replacing PEL with 28 Native American genomes from 13 populations from the Simons Genome Diversity Project (Note that rs7115739 was masked in the SGDP we used data and is not shown).

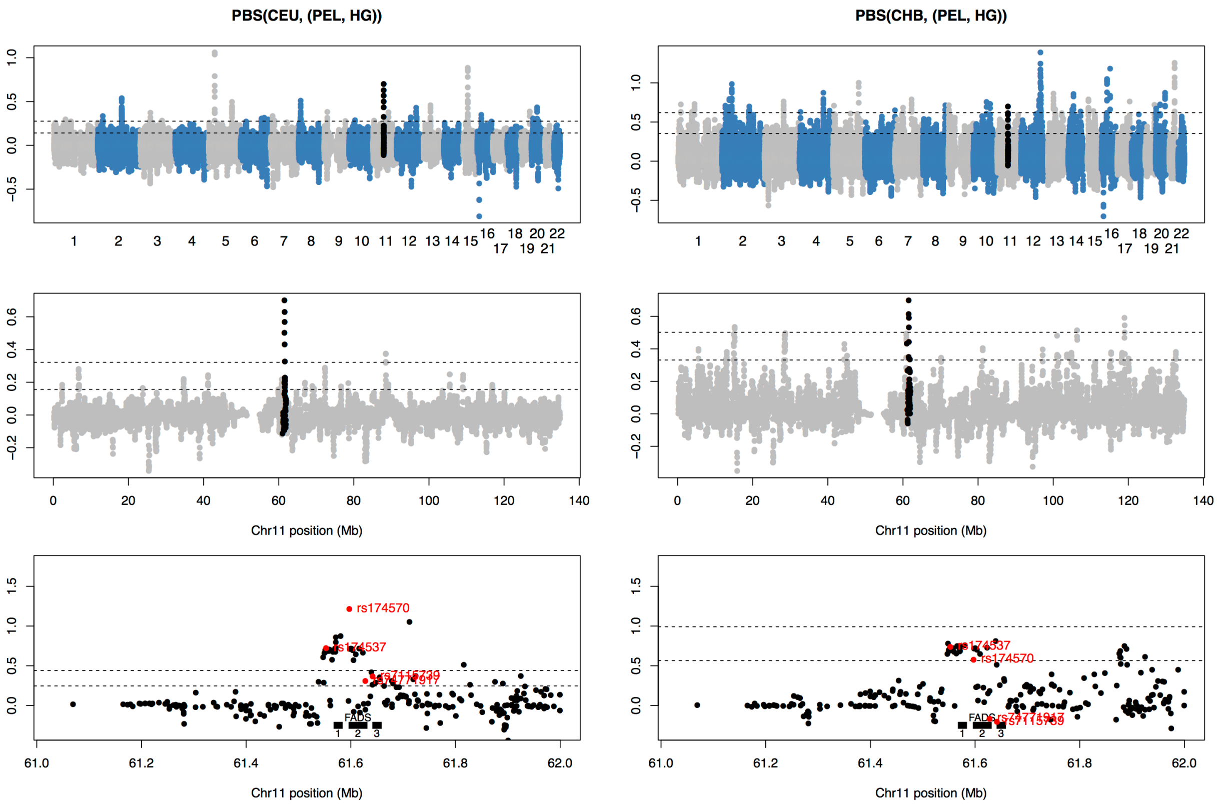

Supplementary Figure 3: As Figure 2 but testing for selection on the CEU (left) and CHB (right) branches, rather than the PEL branch.

CHR	POS	ID	REF	ALT	GoyetQ116	Vestonice 13	Vestonice 16	Ostuni 1	Kostenki 14	Sunghir I	Sunghir II	Sunghir III	Sunghir IV	Afontova Gora 2	Afontova Gora 3	Ust'Ishim	MA1	Tianyuan	Yana	Yana 2
11	61551927	rs174536	A	C	0,1	0,2	0,1	1,1	0,12	0,1	0,4	3,5	0,1	0,0	0,0	0,29	0,1	0,0	11,7	0,8
11	61552680	rs174537	G	T	0,3	0,0	0,0	0,0	0,16	0,1	0,6	3,8	0,2	1,0	0,0	0,19	0,1	0,5	11,10	0,8
11	61557826	rs102274	T	C	0,6	0,3	0,10	1,2	0,93	0,1	0,7	10,7	0,7	0,0	0,0	0,20	0,1	0,11	10,20	0,4
11	61569306	rs174545	C	G	0,0	0,0	0,0	0,0	0,0	0,2	0,5	6,6	0,3	0,0	0,0	0,39	0,0	0,0	14,20	0,8
11	61569830	rs174546	C	T	0,6	0,0	0,17	0,0	0,75	0,0	0,4	7,8	0,7	0,1	0,2	0,38	0,4	0,0	10,14	0,8
11	61570783	rs174547	T	C	0,0	0,0	0,5	0,0	1,19	0,0	0,9	9,3	0,4	0,0	0,0	0,33	0,0	0,7	13,15	1,7
11	61571478	rs174550	T	C	0,0	0,0	0,10	0,0	0,67	0,5	0,5	5,7	0,5	0,1	0,1	0,25	0,1	0,9	11,17	0,6
11	61575158	rs174553	A	G	0,0	0,0	0,0	0,0	0,0	0,0	0,8	3,7	0,3	1,0	0,0	0,30	0,0	0,0	9,17	0,4
11	61579463	rs174554	A	G	0,0	0,0	0,3	1,0	1,20	0,0	0,2	4,4	0,4	0,0	0,0	5,20	0,0	0,0	14,16	1,6
11	61585144	rs174562	A	G	0,0	0,0	0,0	0,0	0,0	0,1	0,4	10,9	0,4	0,0	0,0	1,46	0,0	0,0	21,18	0,3
11	61588305	rs174564	A	G	0,0	0,0	2,0	0,0	7,9	0,0	0,1	3,1	0,5	0,0	0,0	15,15	0,0	0,0	7,1	0,2
**Ancestral (A) or Dervied (D) haplotype**					AA	AA?	AA	AD	AA	AA	AA	AD	AA	AD?	AA?	AA	AA	AA	AD	AA

Supplementary Table 1: Reads supporting the reference (derived) and alternative (ancestral) allele (d,a) at 11 SNPs used to define the derived haplotype (Mathieson and Mathieson 2018).
